# *Streptococcus Sanguis* Biofilm Architecture and Its Influence on Titanium Corrosion in Enriched Artificial Saliva

**DOI:** 10.3390/ma10030255

**Published:** 2017-03-03

**Authors:** Lei Li, Shunling Li, Qing Qu, Limei Zuo, Yue He, Baolin Zhu, Cong Li

**Affiliations:** 1 State Key Laboratory for Conservation and Utilization of Bio-Resources in Yunnan, Yunnan University, Kunming 650091, China; leelei@ynu.edu.cn (L.L.); yajunchenqulee@gmail.com (B.Z.); 2 School of Chemical Science and Technology, Yunnan University, Kunming 650091, China; quqing68@gmail.com (S.L.); 961779457lee@gmail.com (L.Z.); 1201500105@ynu.edu.cn (Y.H.); 3 Public Technical Service Center, Kunming Institute of Zoology, Chinese Academy of Sciences, Kunming 650223, China; licong78@hotmail.com

**Keywords:** titanium, *S. sanguis*, pitting corrosion, artificial saliva, corrosion mechanism

## Abstract

Bacteria biofilm formation on metals is well-known, while biofilm architecture varies under different conditions. To date, few studies have determined the possible contribution to corrosion of titanium made by biofilm architecture. We investigated the interaction between the oral *Streptococcus sanguis* biofilm architecture and its influence on titanium corrosion in enriched artificial saliva using electrochemical methods and microscopic study. Patchy biofilms were observed on titanium surface after being immersed in solution containing *S. sanguis*. The thickness and size of the patchy biofilms increased with an increase of immersion time. The extensive pits were clearly observed by scanning electron microscopy, showing that adsorption of *S. sanguis* on titanium promoted the localized corrosion. The electrochemical results indicated that the corrosion rates were clearly accelerated in the presence of *S. sanguis*. The low *i*_corr_ and high *R*_t_ in the first 48 h indicated that a typical passive behavior still remained. Our study showed that the pitting corrosion of titanium was mainly attributed to the formation of a self-catalytic corrosion cell by the co-effect of patchy biofilm and organic acid secreted by *S. sanguis*.

## 1. Introduction

Titanium and its alloys are widely used in dental implantology and orthopedics due to their good biocompatibility, excellent corrosion resistance and mechanical properties [1,2]. Both high biocompatibility and corrosion resistance can be attributed to the formation of passive film on the surface of titanium and its alloys, consisting mainly of amorphous titanium dioxide (TiO_2_) [3,4]. However, the passive film of titanium and its alloys is often damaged during uses in some complicated environments, such as oral environments. Several studies have been done to describe patients allergic to titanium [5,6,7,8] which were attributed to the corrosion of titanium. Thus, it is important to learn the corrosion behavior and mechanism of titanium in oral environments.

In general, many elements cause the corrosion of titanium in oral environments, but microbially influenced corrosion (MIC) is deemed to be one of the main reasons. Although there are many studies involved in evaluating the corrosion of titanium and its alloys in human physiological solution and oral environment, few investigations have been concerned with the role of oral microorganisms which can form a biofilm on titanium and its alloys in the corrosion process [9,10]. The oral environment is a very complex polymicrobial system, which contains large number of genetically distinct microorganisms [11]. Many of them can colonize the surface of biomaterials and form biofilms. Adhesion of bacterial cells on titanium has been already described both in vivo and in vitro [12,13,14,15,16]. Microbial colonization is initiated by adhesion of pioneer species, such as *Streptococcus oralis*, *Streptococcus sanguis* and *Actinomyces naeslundii*, through interactions with the salivary pellicle [12]. Members of the genus *Streptococci* (including *S. gordonii*, *S. mitis*, *S. oralis* and *S. sanguis*) are observed at many sites in the human oral cavity and numerically abundant in dental plaque [13]. Among these *Streptococci*, *S. sanguis* is one of the predominant organisms and known as a pioneer bacterium [14]. Biofilms of *S. sanguis* not only can grow on glass packed tube (GPT) devices [15], but preferentially colonize the surface of magnetic materials and titanium [16]. Drake et al. [16] confirmed that *S. sanguis* could colonize titanium implants and formed biofilm in vivo. Formation of biofilms on metallic substratum and subsequent bacterial physiological activities change the chemical and electrochemical environment of the metals, such as pH, ion species and strength, oxidation-reduction potential, and the characteristics of passive layer and corrosion products, thus either promoting or inhibiting corrosion [17,18]. So it is necessary to take the influence of oral microorganisms into account when dental materials are evaluated. As a pioneer bacterium in oral materials, the corrosion effect of *S. sanguis* on metals has also drawn much attention [19,20]. Wilson et al. [19] studied the effect of *S. sanguis* on the corrosion of Nd_2_Fe_14_B magnets and pointed out that *S. sanguis* caused appreciable corrosion of Nd_2_Fe_14_B magnets which was greater than that occurring in the absence of the organism. Hu et al. [20] also found that the presence of *S. sanguis* accelerated the corrosion rate of magnetic materials. No pitting corrosion was observed on the magnetic materials’ surface in the presence of *S. sanguis* in previous studies [19,20]. Souza et al. [21] also showed that another strain of streptococcus species, *S. mutans*, could accelerate the corrosion of titanium when immersed in enriched artificial saliva for 2 days. Interestingly, they deemed that the presence of *S. mutans* could form a compact titanium passive film without the occurrence of localized corrosion. These results are clearly different from MIC reported previously, in general, it was believed that the formation of biofilms could promote pitting corrosion [17,18,22]. Therefore, it is necessary to carry out further study to ascertain whether biofilms formed by streptococcus species do not promote localized corrosion. Furthermore, in recent decades, improved microscopic techniques have revealed that a diversity of biofilm structures occur, varying from smooth flat biofilm to forms described as towers, mushrooms, pores and channels, etc. [23,24]. However, there has been no research underlying the bioelectrochemical mechanisms in the presence of *S. sanguis* biofilm structures; therefore, the detailed mechanisms of *S. sanguis* on biomaterials are still poorly understood.

The objective of this investigation is to determine the effect of *S. sanguis* on the corrosion of titanium in enriched artificial saliva. Meanwhile, possible mechanisms are presented to explain the experimental observations.

## 2. Results

### 2.1. Growth Phase

Figure S1 shows the growth phase of *S. sanguis* in the enriched artificial saliva at 37 °C. Four different phases of *S. sanguis* are observed in this figure. In the lag phase, the optical density value at 580 nm (OD_580nm_) increases slowly from 0.02 to 0.10, suggesting the bacteria grow slowly; In logarithmic phase, from 8 to 48 h, the OD_580nm_ value increases quickly from 0.10 to 1.05, showing that the bacteria grow quickly in this stage. In the third stage, there is a plateau from 48 to 72 h corresponding to the maximum growth of the bacteria. After that, the growth of *S. sanguis* enters the decline phase. In this stage, the bacteria die quickly, which can be mainly attributed to the accumulation of metabolite and the consumption of oxygen and nutrients.

### 2.2. pH Results

In order to investigate the effect of *S. sanguis* on the pH value, the pH values of artificial saliva containing working electrodes in the presence and absence of *S. sanguis* are given in Figure S2 at 37 °C for different times. In sterile solution, the pH first increases slightly from 6.70 to 7.60 and then decreases slowly to 7.20, and the solution shows approximate neutrality. However, in the presence of *S. sanguis*, the pH value decreases quickly from 6.70 to 5.40, then it slightly increases with increase of immersion time to 5.70. There is a minimal pH value during the rapid growth period at 24 h which corresponds to the rapid growth stage. The increase of pH after 24 h can be attributed to the anodic dissolution balanced by oxygen-consuming corrosion in the cathodic areas, 2H^+^ + 1/2O_2_ + 2e^−^ ↔ H_2_O. In addition, the pH in the presence of *S. sanguis* is obviously lower than that in the sterile solution, which clearly indicates that *S. sanguis* is responsible for the low pH because *S. sanguis* can secrete organic acid such as lactic acid in their physiological activities [25].

### 2.3. The Bacteria Colonization Study with FM

**Figure 1:**
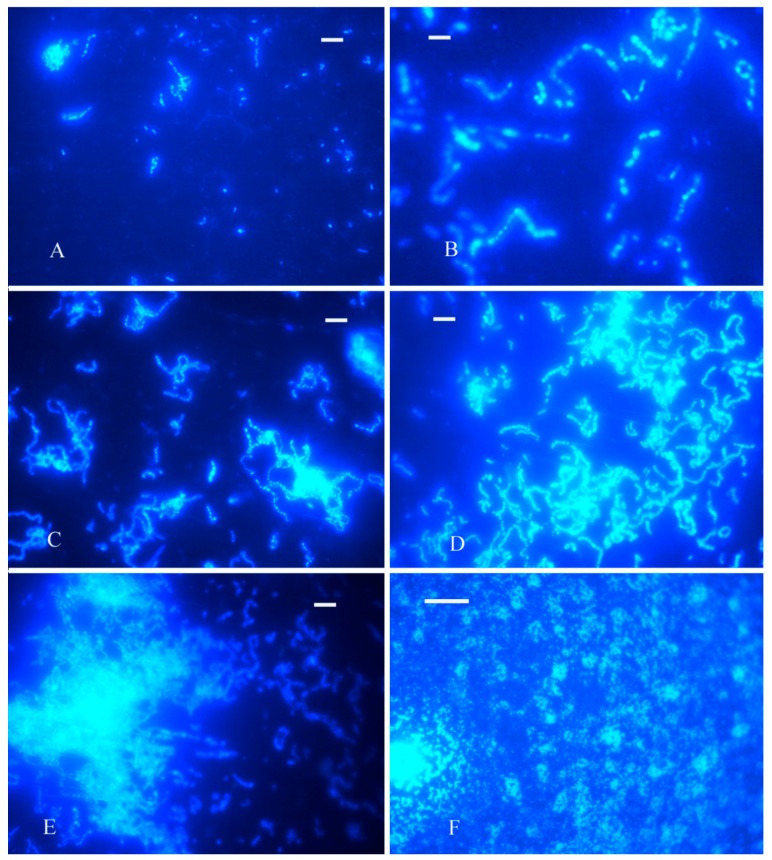
Fluorescence images of bacterial cells colonized titanium specimens in the *S. sanguis* inoculated enriched artificial saliva after various exposure times (**A**) 24 h; (**B**) 72 h; (**C**) 120 h; (**D**) 168 h; (**E**) 336 h; (**F**) magnified view of the local biofilm in (**E**). Scale bar is 50 μm.

Elter et al. [26] proposed the idea that once the clean implant surface was exposed to the oral cavity, it was immediately covered by salivary pellicle and colonized by microorganisms. To demonstrate whether *S. sanguis* can colonize the surface of titanium, fluorescent microscopy (FM) was employed in this study. Figure 1 shows the FM images of *S. sanguis* biofilm development. *S. sanguis* biofilm formed by microcolonies attaches to the experimental titanium surface during constant agitation of the medium at different times. High-resolution imagery shows an uneven distribution of 2-(4-Amidinophenyl)-6-indolecarbamidine dihydrochloride (DAPI) within the biofilm, indicating the staining of nucleic acids within individual cells, whereas zones without cellular structures do not stain positive with DAPI. As it could be clearly seen from Figure 1, *S. sanguis* is spherical or catenulate, and the chains are in different lengths, ranging from 4–5 to 20–30 cells. After titanium is exposed to *S. sanguis* for 24 h, only a small quantity of bacteria can be found and no bacterial clusters appear (Figure 1A). In the subsequent period, the bacteria grow cumulatively and rapidly. With increasing immersion time, more and more bacterial cells adsorb on the titanium surface and aggregate to form bacterial clusters, the patchy biofilm increases in size with increase of immersion time (Figure 1B–E). The maximum level is achieved after 336 h immersion (Figure 1E). A magnified view of local biofilm (Figure 1F), further demonstrates that it is composed of numerous microcolonies but it is not so compact; many channel and lacuna are noticed. This confirms that *S. sanguis* can adhere to the surface of the sample and form the local biofilm.

### 2.4. Surface Topography Analysis with SEM after Conducting Constant Immersion for 336 h

**Figure 2:**
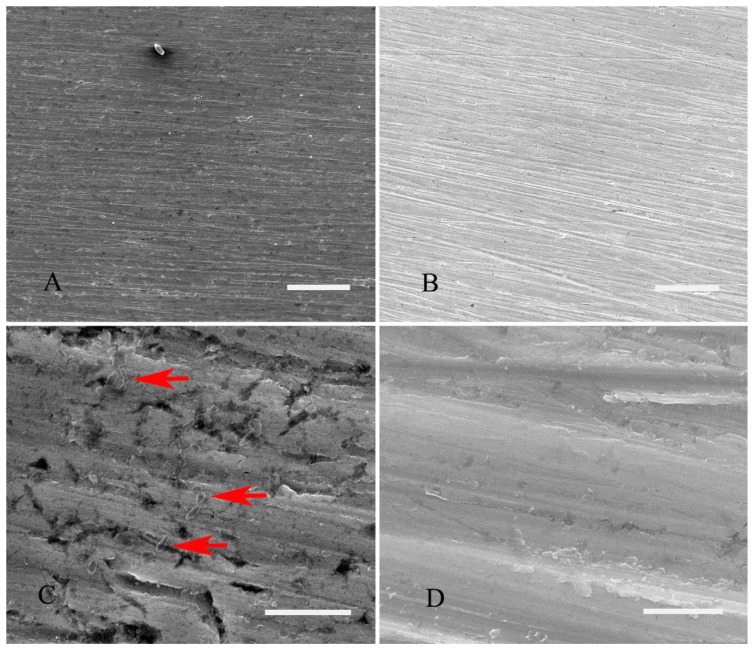
SEM images of titanium in enriched artificial saliva with or without *S. sanguis* for 336 h. (**A**) with *S. sanguis*; (**B**) without *S. sanguis*; (**C**) magnified view of (A) (*S. sanguis* was indicated with red arrows) and (**D**) magnified view of (B). (Scale bar: A, B, 100 μm; C, 10 μm; D, 5 μm).

SEM results after conducting constant immersion for 336 h are presented in Figure 2, which suggests the surface morphology of titanium after being immersed in the media with or without *S. sanguis*. The corrosion surface of titanium looks rather uneven and the titanium surface is covered with a pot-holed layer of deposits. It is clear that more extensive cracks can be observed on the surface of titanium in the presence of *S. sanguis* (Figure 2A)*.* The corrosion of titanium in the absence of *S. sanguis* is inconspicuous compared to that in the presence of *S. sanguis* (Figure 2B). Furthermore, a film presenting a microbial community is also noticed in Figure 2A. Figure 2C is the magnified view of Figure 2A, which further demonstrates that the film is composed of numerous *S. sanguis* cells with abundant extracellular polymeric substances, that is, *S. sanguis* has good adhesion affinities for titanium and adsorption of *S. sanguis* on the surface of titanium can form biofilm. In addition, extensive pits also are clearly found in Figure 2C, which are more serious and bigger than those in the absence of *S. sanguis* (Figure 2D, the magnified view of Figure 2B), the pits are irregular in share and depth. Results indicates that the presence of *S. sanguis* promotes the localized corrosion of titanium.

### 2.5. The Results of OCP

**Figure 3:**
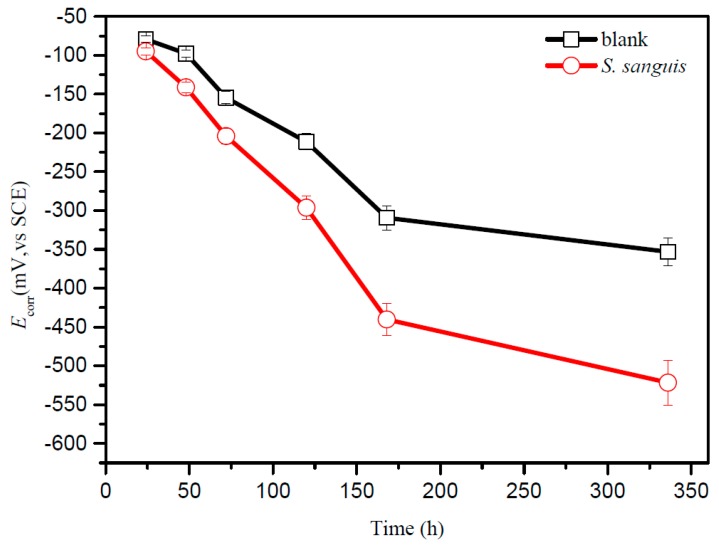
Variation of the open circuit potential (OCP) of titanium in enriched artificial saliva with *S. sanguis* at 37 °C.

The open circuit potential (OCP) recorded on titanium in enriched artificial saliva without or with *S. sanguis* is showed in Figure 3. Both curves in enriched artificial saliva without or with *S. sanguis* have similar shapes, marked diminutions in potential are observed from 24 to 168 h. After 168 h the trends of potential change become smaller. The results also exhibit a clear decrease in *E*_corr_ vs. the saturated calomel electrode (SCE) value with *S. sanguis* compared to that without *S. sanguis*. The similar phenomenon was also observed when the bacteria adsorbed on the surface of other metals [27]. Moradi et al. [27] reported that the adhesion of *Pseudoalteromonas* sp. on the 2205 duplex stainless steel surface in artificial seawater shifted *E*_corr_ vs. SCE to a negative direction and deemed that the phenomenon was attributed to the bacterial activities and metabolic by-products. Thus, in this study the decrease of *E*_corr_ vs. SCE in the presence of *S. sanguis* compared to the sterile solution can be attributed to the following reasons: (a) the adsorption of *S. sanguis* can form biofilm which slows down the diffusion of the cathodic depolarization agents such as oxygen, hydrogen ions, resulting in decrease of the cathodic reduction potential; (b) *S. sanguis* can produce organic acids such as lactic acid, which affect electrochemical process at the titanium surface.

### 2.6. Electrochemical Impedance Spectroscopy (EIS) Studies

**Figure 4:**
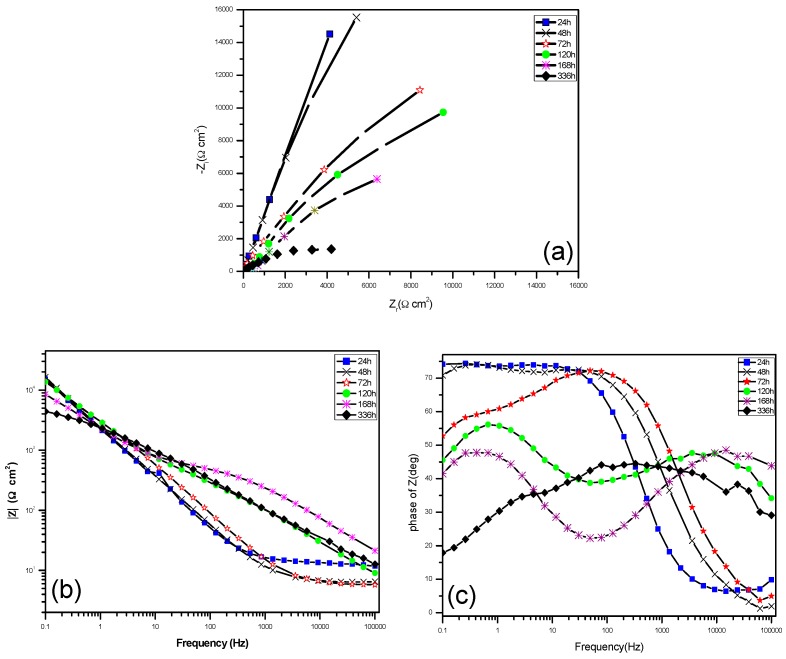
Electrochemical Impedance Spectroscopy (EIS) of titanium immersed for different time in enriched artificial saliva containing *S. sanguis* at 37 °C (**a**) Nyquist plots; (**b**) Bode modulus diagrams; and (**c**) Bode phase angle diagrams.

Figure 4a shows the Nyquist plots of titanium immersed in enriched artificial saliva with *S. sanguis*, it is clearly found that all of the diagrams are a part of the imperfect semicircles and this can be attributed to the frequency dispersion [28,29]. The fact that diagrams have a depressed semicircular appearance shows that the corrosion of titanium is mainly controlled by a charge transfer process. The diagrams show huge values of impedance from 24 to 48 h, suggesting the existence of a passive film, while the values of impedance become smaller after 48 h, which suggests the protective performance of the passive film is gradually weakened. Figure 4b shows the Bode modulus diagrams of titanium immersed in enriched artificial saliva with *S. sanguis*. It is clear that, during the first 48 h, good linearity can be observed in the low and intermediate frequencies and a similar slope is noticed, which suggests a one-time constant related to passive film [10], while two slopes are found in the low and intermediate frequencies after 48 h, which indicates two-time constants. Two-time constants related to a two-layer structure develop during the corrosion [29]. Figure 4c shows the Bode phase angle diagrams, during first 48 h. The impedance spectra exhibit a near capacitive response illustrated by a phase angle close to −80° from medium to low frequency, suggesting that a highly stable film is formed on titanium in the electrolyte used [30]. The phase angle reduces along with the extension of immersing time. After 168 h immersion, two peaks in phase angle plots are noticed, which clearly indicates two relaxation time constants due to a two-layer structure.

According to the above analysis, two equivalent circuits, as shown in Figure S3, are used to verify or rule out mechanistic models and enable the calculation of numerical values corresponding to the electrochemical system under investigation. In the first model (Figure S3a, Model of *R*_s_(*Q*_3_*R*_t_) for 24 h and 48 h immersion coupons), the impedance response is determined by the conductivity of the bulk film and its geometrical capacity. Provided that the mechanism of the passive film does not change significantly with time, the relaxation time for the bulk film will also remain unchanged. However, after 48 h immersion, more and more *S. sanguis* adsorb and form the local biofilm on titanium surface; thus, a second model (Figure S3b, Model of *R*_s_(*Q*_2_*R*_b_) (*Q*_3_*R*_t_) in series with two elements is used for 72, 120, 168 and 336 h immersion coupons). The two elements could be assumed when the metal surface was unpassivated at the moment of its immersion into the electrolyte [31].

In the equivalent circuit, *R*_s_ is the solution resistance; *R*_t_ is the change transfer resistance; *R*_b_ is the film resistance; *Q*_1_ and *Q*_2_ are the constant phase elements (CPEs) parameters for passive film and double layer of film-eletrolyte interface, respectively. The impedance of a phase element is defined as:

(1) $$Q=Z_{\mathrm{CPE}\left(\omega \right)}={\left[{\mathrm{CJ}\omega }^{-n}\right]}^{-1}$$

*n* is a mathematical expression, where −1 ≤ *n* ≤1. The value of *n* is associated with the non-uniform distribution of current as a result of roughness and surface defects. When *n* = 1, the CPEs describe an ideal capacitor [30].

The EIS parameters are fitted by the suggested models and the results are given in Table 1. Results in Table 1 show that *R*_s_ is quite low, *R*_s_ decreases firstly and then increases with the increase of immersion time, which is due to a large number of electrolytes that existed in the enriched artificial saliva. The dissolution of titanium balanced by oxygen-consuming corrosion further increases the electrical conductivity of the solution, but the dissolution amount of titanium decreases with increases in time. Table 1 also shows that *R*_t_ is very large (bigger than 10^5^ Ω∙cm^2^), which is attributed to the passive film on titanium. However, with increase of the immersion time, *R*_t_ decreases rapidly, only several hundred ohm per square centimeter after 48 h immersion, indicating that the corrosion is in an active corrosion process. *R*_b_ shows an increasing trend, which results from the formation of biofilm. This demonstrates that more and more *S. sanguis* adsorb and begin to form biofilm on titanium surface and that the biofilm become thicker with increasing time. Moreover, the value *n*_1_ is higher than 0.80 during the first 72 h, showing a low surface roughness and defect, which is due to the existence of passive film on the surface, but with increasing time, *n*_1_ decreases from 0.80 to 0.50 quickly, which indicates that the passive film is destroyed and becomes increasingly porous and that surface inhomogeneity increases due to the pitting of titanium. *n*_2_ is also decreased with the increase of immersion time.

### 2.7. Potentiodynamic Polarization Curves Tests

**Figure 5:**
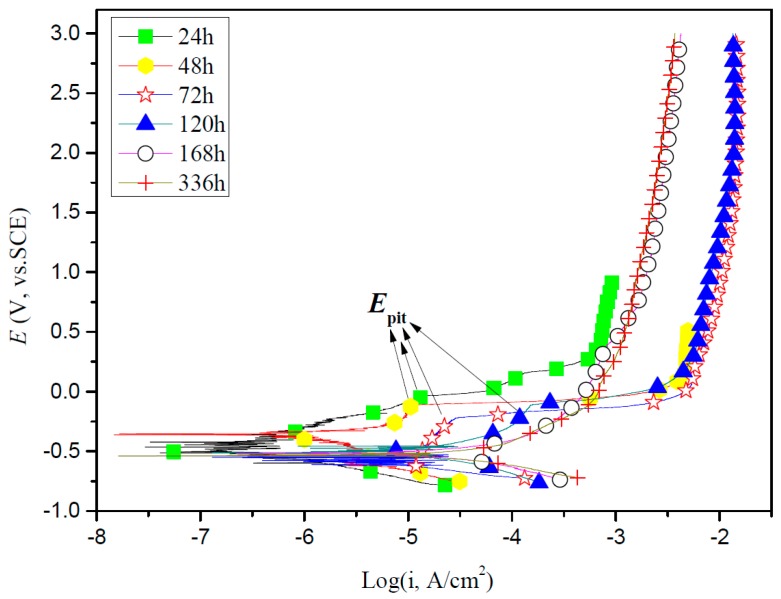
Potentiodynamic polarization curves of titanium exposed to enriched artificial saliva with *S. sanguis* at 37 °C for different time. (*E*_pit_: pitting potential).

Figure 5 presents the potentiodynamic polarization curves results of titanium exposed to enriched artificial saliva with *S. sanguis* for different times. In general, the cathodic branches are shifted to more positive potential direction, while the anodic branches shift to more negative potential direction under the lower potential, which results in a notably increase in *i_corr_*. In addition, the anodic polarization current density increases rapidly with increase in potential after 168 and 336 h immersion; it obviously exhibits an active corrosion characteristic. However, the anodic polarization current density firstly increases slowly or remains unchanged and then a plateau is experienced, with an increase in potential to obtain a certain value during the first 120 h. This suggests that the passive film on titanium still remains but the passive film will be broken while the applied potential is larger than the pitting potential (*E*_pit_). From Figure 5 it is also clear that *E*_pit_ decreases with increase of the immersion time from 24 to 120 h, suggesting that the protective TiO_2_ film on titanium surface is gradually destroyed with increases in the immersion time. Values of the electrochemical corrosion parameters, such as corrosion potential (*E*_corr_), cathodic Tafel slope (*β*_c_), anodic Tafel slope (*β*_a_), corrosion current density (*i*_corr_) are presented in Table 2. From Table 2, we can observe that the corrosion potentials decrease rapidly with increasing immersion time in the initial hours and then tend to be stable. By increasing the immersion time from 24 to 72 h, β_a_ decreases while β_c_ increases. After 72 h of immersion, both β_a_ and β_c_ are irregular. As for *i*_corr_, it increases slowly with increasing immersion time during the first 120 h especially for the first 48 h and then increases remarkably from 168 to 336 h in the enriched artificial saliva medium in the presence of *S. sanguis*, suggesting that the corrosion of titanium in the presence of *S. sanguis* increases with the immersion time and the TiO_2_ film became less protective after long time immersion. These results are in good agreement with the results obtained from EIS.

## 3. Discussion

**Figure 6:**
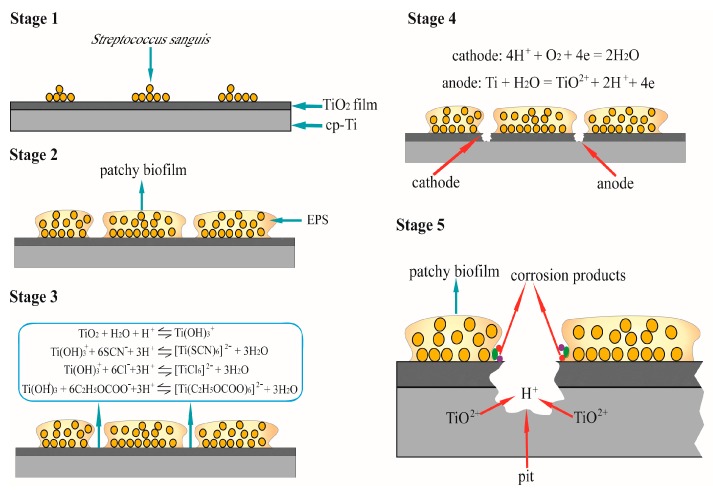
The formation of biofilm and its effect on titanium corrosion: (**Stage 1**) initial attachment of *S. sanguis* cells to the surface of titanium; (**Stage 2**) formation of patchy biofilm; (**Stage 3**) dissolution of TiO_2_ film amongst the patchy biofilms; (**Stage 4**) cathodic and anodic reactions; (**Stage 5**) formation of pit.

The results obtained in this study obviously demonstrate that *S. sanguis* accelerates the corrosion of titanium through the formation of patchy biofilm and the lower pH. As one of the main pioneer bacteria, adhesion of *S. sanguis* cells on oral materials was widely studied [16,19]. Both Drake et al. [16] and Wilson et al. [19] confirmed that *S. sanguis* could also colonize the surface of oral materials and formed biofilm. The formation of biofilm is a long-term process and the major constituents of biofilm are water (70%–90%) and extracellular polymeric substance (EPS) [31]. Microbial EPS are biosynthetic polymers that can be highly diverse in chemical composition and may include substituted and unsubstituted polysaccharides, proteins nucleic acids and phospholipids [32]. It has also been suggested that the free components of EPS released into the bulk phase can compete with the bacterial cells for binding sites on the metal surface, thus further contributing to the bio-corrosion [33]. Dexter [34] showed that it took about 3 to 7 days for the biofilm to form on SS316 and SS304 surfaces in natural seawater. Heydorn et al. [35] showed that biofilm of *Pseudomonas* species become mature after 10 days. Our study also shows that fewer *S. sanguis* attach to the surface of titanium at the beginning, but the adsorption quantity of bacteria increase with increasing culture time, then the local biofilm begins to form. After 336 h, a mature local biofilm is noticed. Magnified view of the local biofilm further demonstrates that it is composed of numerous bacterial microcolonies. The formation of biofilm and its effect on titanium are illustrated in Figure 6. Stage 1 in Figure 6 is the initial attachment of cells to the surface of titanium due to the higher surface energy of the protective and self-adherent oxide film, then bacteria produce EPS, which results in more firmly adhered “irreversible” attachment. The connection between EPS formation and differentiated biofilm structure is supported by Danese et al. [36] and Watnick et al. [37], who reported that colonic acid and EPS were required for the development of complex structures in bacteria biofilm. In the stage 2, the bacteria grow cumulatively and rapidly. With increasing the immersion time, more and more bacteria cells adsorb on the titanium surface and aggregate to form a patchy biofilm. The patchy biofilm increases in thickness and size with increase of immersion time. The formation of patchy biofilm may change the condition of the environment, and the biofilm matrix forms a transport barrier, which may prevent the penetration of corrosive agents, resulting in a decrease in OCP with increasing the immersion time (Figure 3). Similar results have been also observed by Jack et al. [38], they deemed that the biofilm formation and electrode colonization were responsible for the decrease of OCP for carbon steel. Qu et al. [39] also suggested that biofilm formation resulted in more evident decrease of the OCP value in the presence of bacteria in their study. In the areas where there was no bacteria colonization, TiO_2_ film still was exposed to enriched artificial saliva. Due to the formation of relatively stable complex, the dynamic balance between dissolution and reparation of passive film is likely to be destroyed by the small anions in solution such as Cl^−^, SCN^−^ etc. [10,40,41] (see the stage 3 in Figure 6). The destruction of TiO_2_ film results in the direct exposure of titanium substrate to solution. The exposed titanium areas became anodic areas with other areas below the colonies becoming cathodic, which forms a galvanic corrosion (stage 4), thus promoting pitting corrosion (stage 5). The corrosion is mainly concentrated in the pits, the surface integrality of the areas around the pits is better due to a certain cathodic polarization. SEM results in our study clearly confirm that pitting corrosion in the presence of *S. sanguis* occurs, and this form of corrosion is more serious than that in the absence of *S. sanguis*. That is, adsorption of *S. sanguis* on titanium promotes the localized corrosion. Although localized corrosion of materials in the presence of biofilm was also reported in the previous studies [18,27,33], it is very interesting that Souza et al. [21,42] argued that they did not detected localized corrosion on titanium after colonization with single or mixed biofilms. They deemed that the formation of a compact passive film on titanium in the presence of *streptococcus* species should be responsible for general corrosion. However, our study supports the proposal that the patchy biofilms are formed on titanium and responsible for the localized corrosion.

Furthermore, the presence of biofilm and bacterial activity can contribute to the deterioration of passivity by accumulation of organic acid, such as lactic acid at the localized areas. Svensater et al. [25] found that the *S. sanguis* could metabolize lactate and acetate in both anaerobic and aerobic conditions. In fact, lactic acid also is one of the uppermost factors in the mouth, which may affect the corrosion of the metal. Koike and Fujii [43] reported that titanium was dissolved and became discolored in contact with lactic acid or formic acid in physiological saline and enriched artificial saliva by immersion tests. Takahashi et al. [44] evaluated the corrosion resistance of Ti-Ag alloys in a 1% lactic acid solution. They showed that the Ti-Ag alloys had excellent corrosion resistance that was comparable or superior to that of pure titanium. Koike and Fujii [45] declared that the corrosive properties of titanium were markedly dependent on pH in formic acid and relatively less dependent on pH in lactic acid. The results of Qu et al. [10] showed that the corrosion of titanium was distinctly affected by lactic acid, and the corrosion rate increased with increasing amount of lactic acid. The role of lactic acid can be attributed to two aspects: acidification of the solution and formation of a chelate compound by lactic acid (the stage 3 in Figure 6) that both destroy the dynamic balance between dissolution and reparation of the passive film. In this study, it is clear that pH in the presence of *S. sanguis* is obviously lower than that in the sterile solution; thus, *S. sanguis* is responsible for the lowered pH, which forms a self-catalytic corrosion environment and results in the dissolution of titanium at the anodic areas (stage 5 in Figure 6). Previous studies also revealed that the pH in the biofilm could be much lower than in the bacteria-free system and acid production by the biofilms might have contributed to the corrosion [39]. Therefore, the micro-organisms colonized the surface by forming a biofilm produce an environment at the metal/biofilm interface which is different from the initial general medium (pH, dissolved oxygen content and organic/inorganic species) [46]. The changed environment further accelerates the dissolution of the protective and self-adherent oxide film on titanium. That is, lactic acid generated by *S. sanguis* in the solutions can attack the titanium surface before and after formation of the biofilm, which leads to the tendency toward pitting corrosion. The results obtained from polarization curves and EIS also show that the corrosion increases with increasing of the bacteria adsorption, which is related to an increase of *i*_corr_ and decrease of *R*_ct_ with increase of the immersion time. These results prove the unquestionable influence of *S. sanguis* on the corrosion behavior of titanium in enriched artificial saliva by formation of biofilm and acid metabolite. Therefore, the role of oral microorganisms in the corrosion process should be considered when new alloys are being evaluated as dental materials.

## 4. Materials and Methods

### 4.1. Preparation of Test Materials

The experiment were performed with medical grade pure titanium with the following chemical composition (wt %): Ti ≥ 99.405, Fe ≤ 0.25, C ≤ 0.10, N ≤ 0.03, H ≤ 0.015, O ≤ 0.20.

For the electrochemical measurements, the working electrode was made of the pure titanium specimen in polyvinyl chloride (PVC) holder using epoxy resin with an exposed area of 1.0 × 1.0 cm^2^. For the fluorescent microscopy and scanning electron microscopy measurement, a series of pure titanium tablets (20 × 20 × 1 mm^3^) were prepared. All of the specimens were abraded with silicon carbide (SiC) abrasive paper from 120 to 2000 # grades on the test surface, rinsed with distilled water, degreased with acetone (CH_3_COCH_3_), sterilized with 2.5% glutaraldehyde solution for 2 h and then exposed in UV light for 15 min for sterilization. After sterilization the specimens were directly used as the working electrodes for the test.

### 4.2. Bacterium

The strain of *S. sanguis* (CGMCC No. 1.2497) was originally obtained from the China General Microbiological Culture collection Center (CGMCC, Beijing, China), and cultured in brain heart infusion broth (BHI) agar at 37 ± 2 °C.

### 4.3. Medium and Solution

All tests were conducted using enriched artificial saliva containing Na_2_HPO_4_: 0.26 g/L, NaCl: 6.7 g/L, KSCN: 0.33 g/L, KH_2_PO_4_: 0.2 g/L, NaHCO_3_: 1.5 g/L, KCl: 1.2 g/L, carbamide: 1.5 g/L, BHI: 37 g/L. The pH of the medium was adjusted to 6.7 using 0.1 M hydrochloric acid (HCl) solution and sterilized by autoclaving at 121 °C and at 100 kPa for 20 min.

### 4.4. Growth Phase Experiment

Growth phase experiments were performed to determine the growth kinetics of *S. sanguis* in enriched artificial saliva at 37 °C. A loop of *S. sanguis* cell from a slant culture of fresh nutrient agar was inoculated to a 250 mL Erlenmeyer flask containing 100 mL BHI broth. The flask was incubated on a rotary shaker at 150 rpm at 37 °C for 24 h until the optical density of the culture at 580 nm (OD_580nm_) reached about 1.0, then 1 mL bacterial culture was taken out by sterile pipette and inoculated into a volume of 150 mL enriched artificial saliva in 250 mL flask. The flask was incubated with a stirring speed of 150 rpm using a polytetrafluoroethylene magnetic stirring at 37 °C by a thermostatically controlled water tank. In this process, three parallel flasks were performed. During incubation, 1 mL culture from each of three parallel flasks was collected and pooled every 4 h, the combined samples were then subjected to cell density measurement. OD_580nm_ was measured over time using a photometer. The experiment was terminated until bacterial cells reached the decline phase. All experiments were performed in triplicate.

### 4.5. Fluorescent Microscopy (FM)

To determine the biofilm growth on the titanium surface, the treated pure titanium tablets were exposed to the *S. sanguis* inoculated medium during constant agitation without renewing the fresh medium for 24, 48, 72, 120, 168 and 336 h, respectively. At the predetermined period of bacterial incubation, the specimens were retrieved and transferred to plate containing 0.1 M phosphate buffered saline (PBS) to remove the dead and loosely attached bacteria. This process was repeated for 3 times. Then the specimens were stained with 2-(4-Amidinophenyl)-6-indolecarbamidine dihydrochloride (DAPI) solution for 15 min. The specimens with bacteria cells were imaged under 20× and 40× magnifications using a Nikon E800 fluorescence microscope (Nikon; Tokyo, Japan), equipped for epifluorescence with a mercury lamp.

### 4.6. Surface Analysis by Scanning Electron Microscopy (SEM)

After conducting constant immersion in enriched artificial saliva with and without *S. sanguis* for 336 h, the samples were taken out. Prior to characterizing the morphologies of corrosion, the samples were carefully washed with PBS solution, then fixed by 15 min immersed in 2.5% glutaraldehyde, dehydrated in a series of aqueous ethanol solutions (15%, 30%, 50%, 70%, 95% and 100% *v*/*v*). Then the surface morphology was examined by scanning electron microscopy (FSEM-TMPFEI Quanta 200, FEI, Columbus, OH, USA).

### 4.7. pH Tests

pH tests were also performed in enriched artificial saliva solutions containing a working electrode with and without *S. sanguis* before electrochemical experiments. pH tests were surveyed by PHS-25 (Hongyi Instrument Company, Shanghai, China) and performed in triplicate.

### 4.8. Electrochemical Experiments at Different Times

The electrochemical measurements were carried out in an Erlenmeyer flask using a potentiostat of the PARSTAT 2263 electrochemical tester (Perkin Elmer™ Company, Boston, MA, USA) interface to a personal computer. A three-electrode system including a working electrode, an auxiliary electrode and a reference electrode was used for electrochemical measurements. The working electrode was the pretreated titanium specimen. The auxiliary electrode was a platinum foil and the reference electrode was a saturated calomel electrode (SCE) with a Luggin capillary positioned close to the working electrode surface in order to minimize ohmic potential drop. EIS measurements were conducted at the end of open circuit potential (OCP) measurement to ensure it was in steady and carried out in a frequency range of 0.1–10^5^ Hz using a 10 mV peak-to-peak voltage excitation. After EIS measurements, the Potentiodynamic polarization was carried out on the same samples by polarizing in the range between −250 and +2500 mV with respect to *E*_corr_ vs. SCE at a scan rate of 1 mV/s. All experiments were performed in enriched artificial saliva solution with a stirring speed of 150 rpm using a polytetrafluoroethylene magnetic stirring with or without *S. sanguis*. The test temperature was kept at 37 °C for the whole test duration by means of a thermostatically controlled water tank. Each experiment was repeated at least three times to ensure the reproducibility.

## 5. Conclusions

Corrosion of titanium in the presence of *S. sanguis* was investigated using surface analysis and electrochemical techniques. The main conclusions can be drawn as follows:

(1) *S. sanguis* can adhere to the surface of titanium and the adsorption of bacteria leads to the formation of local biofilm. The biofilm increased in thickness and size with increasing the immersion time.
(2) The possible contribution of *S. sanguis* to corrosion of titanium cannot be ignored. Adsorption of *S. sanguis* on titanium, especially when growing as biofilm, promotes the localized corrosion.
(3) Results of the electrochemical techniques clearly show that *S. sanguis* can decrease the *R*_t_ and *E*_corr_ but increase the *i*_corr_, indicating that the corrosion rate is clearly accelerated in the presence of *S. sanguis.*
(4) pH is decreased in the presence of *S. sanguis*, which forms a self catalytic corrosion environment and results in the dissolution of titanium at the anodic areas, thus, accelerates the corrosion rate and gives rise to the localized corrosion.
(5) The corrosion of titanium is the co-effect of lactic acid secreted by *S. sanguis* and biofilm formed by *S. sanguis,* both the biofilm and acid metabolite accelerate the dissolution of passive film (TiO_2_) and lead to a tendency of the localized corrosion.

## Figures and Tables

**Table 1:** Analysis of electrochemical impedance spectroscopy (EIS) for titanium in enriched artificial saliva with *S. sanguis* at 37 °C for different exposure times.

t	*R*_s_	*Q*_2_	*n*_2_	*R*_b_	*Q*_1_	*R*_t_	*n*_1_
(h)	(Ω∙cm^2^)	(Ω^−1^∙cm^−2^∙s^n^)	-	(Ω∙cm^2^)	(Ω^−1^∙cm^−2^∙s^n^)	(×10^5^ Ω∙cm^2^)	-
24	12.82	-	-	-	91.97	3.24	0.8944
48	6.30	-	-	-	99.36	2.67	0.8955
72	5.72	7.224	0.8180	494.7	102.40	1.13	0.8560
120	19.03	10.49	0.7827	395.3	107.99	0.41	0.7431
168	68.57	11.70	0.6940	529.3	119.10	0.35	0.6183
336	74.76	15.48	0.5038	609.8	122.54	0.23	0.5832

**Table 2:** Tafel parameters of polarization curves for titanium in enriched artificial saliva with *S. sanguis* at 37 °C for different exposure times.

t (h)	*E*_corr_ (mV vs. SCE)	*i*_corr_ (μA/cm^2^)	*β*_a_ (mV/degree)	*β*_c_ (mV/degree)
24	−395	0.55	220	−193
48	−425	0.99	158	−163
72	−434	1.21	178	−157
120	−524	1.74	369	−525
168	−533	2.02	223	−230
336	−550	4.01	247	−427

## Supplementary Materials

The following are available online at www.mdpi.com/1996-1944/10/3/255/s1. Figure S1: The growth phase of *S. sanguis* in sterile artificial saliva at 37 °C, Figure S2: pH value of artificial saliva containing working electrode with and without S. sanguis at 37 °C for different time, Figure S3: Equivalent circuits used for fitting the impedance spectra.

## References

[1] Mabilleau G., Bourdon S., Joly-Guillou M.L., Filmon R., Baslé M.F., Chappard D. (2006). Influence of fluoride, hydrogen peroxide and lactic acid on the corrosion resistance of commercially pure titanium. Acta Biomater..

[2] Harzer W., Schröter A., Gedrange T., Muschter F. (2001). Sensitivity of titanium brackets to the corrosive influence of fluoride-containing toothpaste and tea. Angle Orthod..

[3] Tengvall P., Lundström I. (1992). Physico-chemical considerations of titanium as a biomaterial. Clin. Mater..

[4] Pan J., Leygraf C., Thierry D., Ektessabi A.M. (1997). Corrosion resistance for biomaterial applications of TiO_2_ films deposited on titanium and stainless steel by ion-beam-assisted sputtering. J. Biomed. Mater. Res..

[5] Urban R.M., Jacobs J.J., Tomlinson M.J., Gavrilovic J., Black J., Peoc’h M. (2000). Dissemination of wear particles to the liver, spleen, and abdominal lymph nodes of patients with hip or knee replacement. J. Bone Jt. Surg. Am..

[6] Lalor P.A., Revell P.A., Gray A.B., Wright S., Railton G.T., Freeman M.A. (1991). Sensitivity to titanium, a cause of implant failure?. Bone Jt. J..

[7] Basketter D.A., Whittle E., Monk B. (2000). Possible allergy to complex titanium salt. Contact Dermat..

[8] Yamauchi R., Morita A., Tsuji T. (2000). Pacemaker dermatitis from titanium. Contact Dermat..

[9] Hanawa T., Asami K., Asaoka K. (1998). Repassivation of titanium and surface oxide film regenerated in simulated bioliquid. J. Biomed. Mater. Res..

[10] Qu Q., Lei W., Chen Y., Li L., He Y., Ding Z. (2014). Corrosion behavior of titanium in enriched artificial saliva by lactic acid. Materials.

[11] Paster B.J., Olsen I., Aas J.A., Dewhirst F.E. (2000). The breadth of bacterial diversity in the human periodontal pocket and other oral sites. Periodontology.

[12] Kolenbrender P.E., Pamler R.J., Periasamy S., Jakubovics N.S. (2010). Oral multispecies biofilm development and the key of cell-cell distance. Nat. Rev. Microbiol..

[13] Tagger G.N., Machtei E.E., Horwitz J., Peled M. (2002). Fracture of dental implants: Literature review and report of a case. Implant Dent..

[14] Del-Curto B., Brunella M.F., Giordano C., Pedeferri M.P., Valtulina V., Visai L., Cigada A. (2005). Decreased bacterial adhesion to surface-treated titanium. Int. J. Artif. Organs..

[15] Embleton J.V., Newman H.N., Wilson M. (1998). Influence of growth model and sucrose on susceptibility of *Streptococcus sanguis* to amine fluorides and amine fluoride-inorganic fluoride combinations. Appl. Environ. Microbiol..

[16] Darke D.R., Paul J., Keller J.C. (1990). Primary bacterial colonization of implant surfaces. Int. J. Oral Maxillofac. Implants..

[17] Videla H.A., de Mele M.F.L., Brankevich G. (1988). Technical note: Assessment of corrosion and microfouling of several metals in polluted seawater. Corrosion.

[18] Mansfeld F. (2007). The interaction of bacteria and metal surfaces. Electrochim. Acta.

[19] Wilson W., Kpendema H., Noar J.H., Hunt N., Mordan N.J. (1995). Corrosion of intra-oral magnets in the presence and absence of biofilm of *Streptococcus sanguis*. Biomaterials.

[20] Hu W.Q., Chen L.D., Wang Y.J., Sun D.F., Han D.W. (2008). The influence of *Streptococcus sanguis* on corrosion resistance of magnetic retainer. Shanghai J. Stomatol..

[21] Souza J.C.M., Ponthiaux P., Henriques M., Oliveira R., Teughels W., Celis J.P., Rocha L.A. (2013). Corrosion behaviour of titanium in the presence of *treptococcus mutans*. J. Dent..

[22] Cheng S., Tian J.T., Chen S.G., Lei Y.H., Chang X.T., Liu T., Yin Y.S. (2009). Microbially influenced corrosion of stainless steel by marine bacterium V*ibrio natriegens*:(I) Corrosion behavior. Mater. Sci. Eng. C.

[23] Fowler A.C., Kyrke-Smith T.M., Winstanley H.F. (2016). The development of biofilm architecture. Proc. R. Soc. A.

[24] Flemming H.C., Wingender J. (2010). The biofilm matrix. Nat. Rev. Microb..

[25] Svensäter G., Takahashi-Abbe S., Abbe K., Birkhed D., Yamada T., Edwardsson S. (1985). Anaerobic and aerobic metabolism of sorbitol in *Streptococcus sanguis* and *Streptococcus mitior*. J. Dent. Res..

[26] Elter C., Heuer W., Demling A., Hannig M., Heidenblut T., Bach F.W., Stiesch-Scholz M. (2008). Supra-and subgingival biofilm formation on implant abutments with different surface characteristics. Int. J. Oral Maxillofac. Implants.

[27] Moradi M., Song Z., Yang L., He J. (2014). Effect of marine *Pseudoalteromonas* sp. on the microstructure and corrosion behaviour of 2205 duplex stainless steel. Corros. Sci..

[28] Mansfeld F. (2003). The use of electrochemical techniques for the investigation and monitoring of microbiologically influenced corrosion and its inhibition. Rev. Mater. Corros..

[29] Lebrini M., Laggrenee M., Vezin H., Traisnel M., Bentiss F. (2007). Experimental and theoretical study for corrosion inhibition of mild steel in normal hydrochloric solution by some new macrocylic polyther compounds. Corros. Sci..

[30] Assis S.L.D., Stephan W., Isolda C. (2006). Corrosion characterization of titanium alloys by electrochemical techniques. Electrochim. Acta.

[31] Stoodley P., Sauer K., Davies D.G., Costerton J.W. (2002). Biofilms as complex differentiated communities. Annu. Rev. Microbiol..

[32] Wigebder J., Neu T.R., Flemming H.C. (1999). What are bacterial exteacellular polymeric substances?. Microbial Extracellular Polymeric Substances.

[33] Breur H.J.A., Wit J.H.W., Van Tumhout J., Ferrari G.M. (2002). Electrochemical impedance study on the formation of biological iron phosphate layers. Electrochim. Acta.

[34] Wang W., Wang J., Li X., Xu H., Wu J. (2004). Influence of biofilm growth on corrosion potential of metal immersed in seawater. Mater. Corros..

[35] Heydorn A., Nielsen A.T., Hentzer M., Sternberg C., Givskov M., Ersbøll B.K., Molin S. (2000). Quantification of biofilm structures by the novel computer program COMSTAT. Microbiology.

[36] Danese P.N., Partt L.A., Kolter R. (2000). Exopolysaccharide production is required for development of Escherichia coli K-12 biofilm architecture. J. Bacteriol..

[37] Watnick P.I., Lauriano C.M., Klose K.E., Croal L., Kolter R. (2001). The absence of a flagellum leads to altered colony morphology, biofilm development and virulence in *Vibrio cholerae* O139. Mol. Microbiol..

[38] Jack R.F., Ringerberg D.B., White D.C. (1992). Differential corrosion rate of carbon steel by combinations of *Bacillus* sp., *Hafina alvei* and *Desulfovibrio gigas* established by phospholipids’ analysis of electrode biofilm. Corros. Sci..

[39] Qu Q., Hea Y., Wanga L., Xua H., Lib L., Chena Y., Dinga Z. (2015). Corrosion bahavior of cold rolled steel in artificial seawater in the presence of *Bacillus subtilis* C2. Corros. Sci..

[40] Fekry A.M. (2009). The influence of chloride and sulphate ions on the corrosion behavior of Ti and Ti-6Al-4V alloy in oxalic acid. Electrochim. Acta.

[41] Misiuk W. (2005). Extractive spectrophotometric methods for the determination of doxepin hydrochloride in pharmaceutical preparations using titanium (IV) and iron (III) thiocyanate complexes. Farmaco.

[42] Souza J.C., Henriques M., Oliverira R., Teughels W., Celis J.P. (2010). Do oral biofilms influence the wear and corrosion behavior of titanium?. Biofouling.

[43] Iwami Y., Yamada T. (1985). Regulation of glycolytic rate in *Streptococcus sanguis* grown under glucose-limited and glucose-excess conditions in a chemostat. Infect. Immun..

[44] Koike M., Fujii H. (2001). In vitro assessment of corrosive properties of titanium as a biomaterial. J. Oral Rehabil..

[45] Masatoshi T., Masafumi K., Yukyo T. (2011). Corrosion behavior of Ti-Ag alloys used in dentistry in lactid acid solution. Met. Mater. Int..

[46] Laurent F., Grosgogeat B., Reclaru L., Dalard F., Lissac M. (2000). Comparison of corrosion behaviour in presence of oral bacteria. Biomaterials.

