# Supplementary material for: Streptococcus Sanguis Biofilm Architecture and Its Influence on Titanium Corrosion in Enriched Artificial Saliva
Source: Materials (Basel). 2017 Mar 3;10(3):255. doi: 10.3390/ma10030255 (PMC5503383; doi:10.3390/ma10030255)
Supplement: Supplementary file 1 [file materials-10-00255-s001.pdf]

# Supplementary Materials: *Streptococcus Sanguis* Biofilm Architecture and Its Influence On Titanium Corrosion in Enriched Artificial Saliva

**Lei Li, Shunling Li, Qing Qu, Limei Zuo, Yue He, Baolin Zhu, Yaxin Kang, Xingcan Qian, Xin Ran and Cong Li**

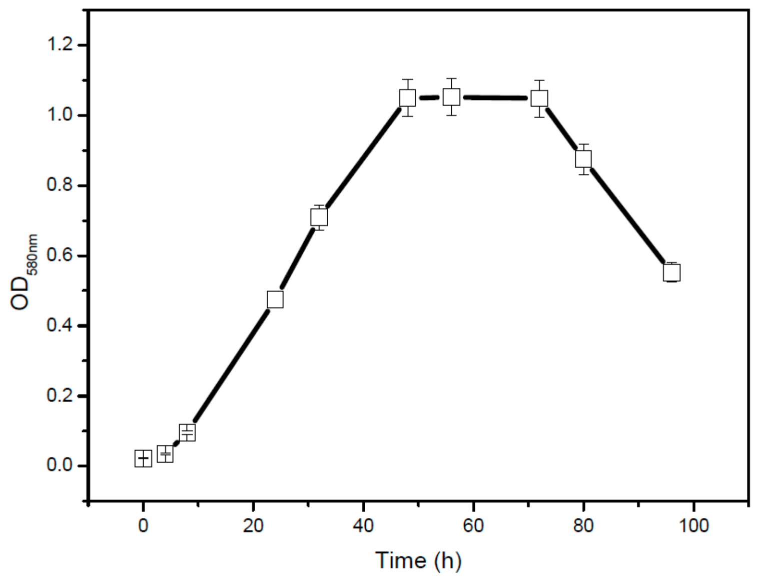


**Figure S1.** The growth phase of *S. sanguis* in sterile artificial saliva at 37 °C.

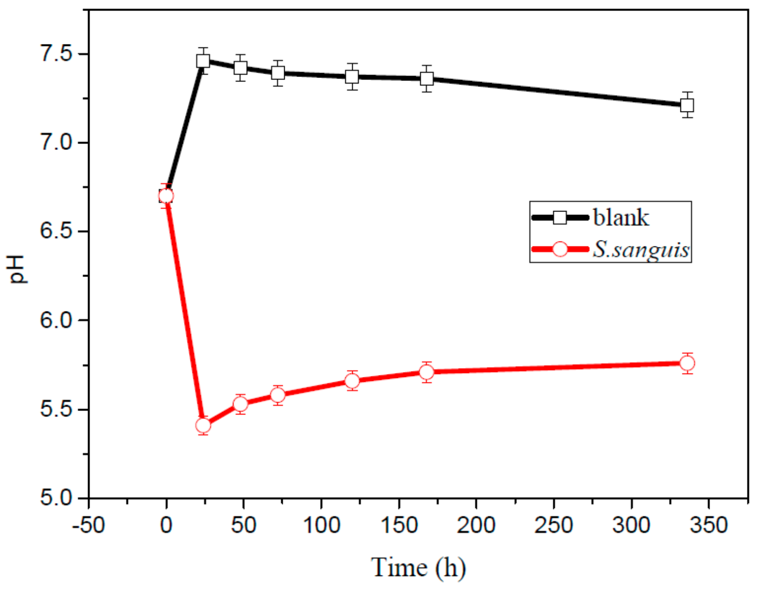


**Figure S2.** pH value of artificial saliva containing working electrode with and without *S. sanguis* at 37 °C for different time.

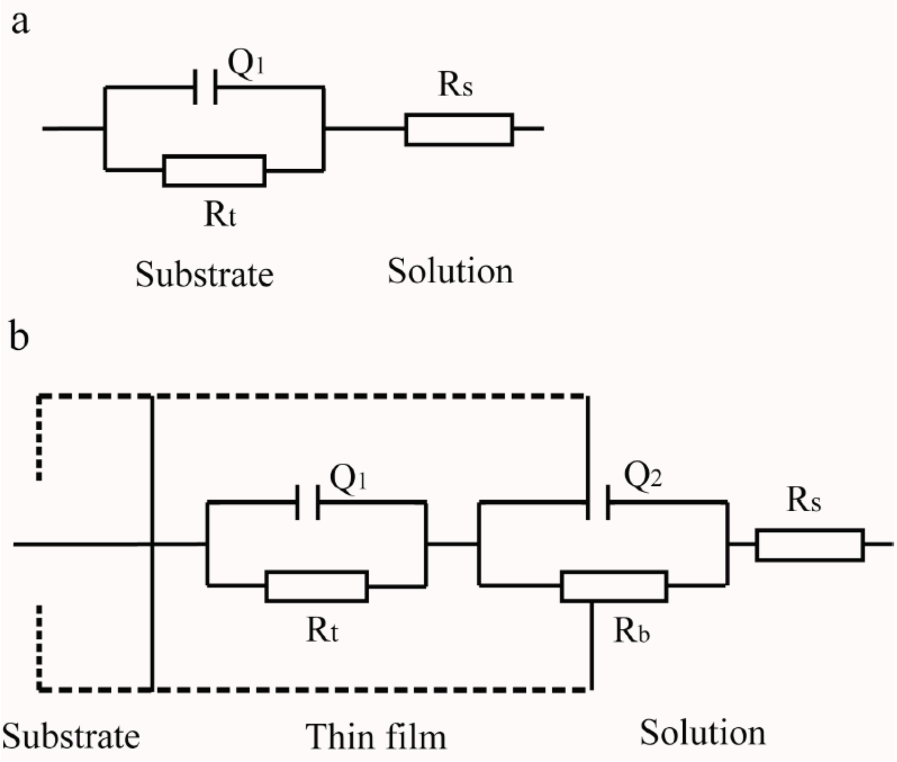


**Figure S3.** Equivalent circuits used for fitting the impedance spectra. (**a**) Model of $R_s(Q_1R_t)$ for 24 and 48 h immersion coupons; (**b**) Model of $R_s(Q_2R_b)$ $(Q_1R_t)$ for 72, 120, 168 and 336 h immersion coupons.

© 2017 by the authors. Submitted for possible open access publication under the terms and conditions of the Creative Commons Attribution (CC-BY) license (http://creativecommons.org/licenses/by/4.0/).
